# Enterococcus faecalis Endocarditis as a Diagnostic Clue to Occult Rectal Cancer: A Two-Case Series

**DOI:** 10.7759/cureus.114209

**Published:** 2026-08-09

**Authors:** Hiba Rizkou, Joumana Elmasrioui, Jihane Rizkou, Khadija Krati, Mustapha El Hattaoui

**Affiliations:** 1 Cardiology, Mohammed VI University Hospital Center, Marrakech, MAR; 2 Physiology, Faculty of Medicine and Pharmacy, Cadi Ayyad University, Marrakech, MAR; 3 Gastroenterology and Hepatology, Mohammed VI University Hospital Center, Marrakech, MAR

**Keywords:** bacteremia, colorectal neoplasia, enterococcus faecalis, gastrointestinal portal of entry, infective endocarditis, rectal cancer

## Abstract

Enterococcus faecalis is an important cause of bacteremia and infective endocarditis, but the portal of entry often remains unclear. Enterococcal endocarditis is potentially fatal and predominantly affects older men in published cohorts. We describe two men, aged 69 and 40 years, who presented with E. faecalis infective endocarditis and gastrointestinal symptoms. The first patient was admitted with fever and ischemic stroke after three months of chronic diarrhea. Transthoracic echocardiography demonstrated a 12.5 × 5.6 mm mitral-valve vegetation. Pelvic magnetic resonance imaging and colonoscopy revealed a stenosing mid-rectal tumor, and biopsy confirmed invasive rectal adenocarcinoma staged as cT4aN2b. The second patient presented with fever, polyarthritis, and bloody diarrhea. Echocardiography showed a 13.2 × 6.5 mm aortic-valve vegetation, while computed tomography and colonoscopy revealed an ulcerated upper rectal mass with histopathological confirmation of mucinous adenocarcinoma. Both isolates were susceptible to vancomycin and gentamicin, and both patients received six weeks of intravenous vancomycin plus gentamicin. Follow-up blood cultures became negative in both cases. In Case 1, C-reactive protein decreased from 359 to 60 mg/L, transient renal dysfunction associated with glomerulonephritis resolved, and neurological recovery after ischemic stroke was partial. In Case 2, C-reactive protein normalized. Follow-up transthoracic echocardiography showed marked improvement after antimicrobial therapy. These cases support targeted colorectal evaluation in selected patients with E. faecalis endocarditis, particularly when gastrointestinal symptoms are present or no alternative portal of entry is identified.

## Introduction

Enterococcus faecalis is a Gram-positive commensal of the human gastrointestinal tract and an important opportunistic pathogen. Its ability to adhere to host tissues, form biofilms, and express multiple virulence factors contributes to persistent bacteremia and infective endocarditis [1]. Enterococcal infective endocarditis is potentially lethal and requires prompt microbiological, echocardiographic, and clinical assessment because it may be complicated by embolic events, valvular destruction, heart failure, septic shock, and immune-mediated manifestations [2,3]. In a prospective international cohort of 500 patients with enterococcal endocarditis, the mean age was 65 years, 72.2% were men, and the one-year mortality rate was 28.9% [3]. A contemporary multicenter cohort similarly showed a median age of 72 years and a male predominance of 69.2% [4].

When no urinary, biliary, catheter-related, or recent procedural source is identified, the gastrointestinal tract should be considered as a possible portal of entry. Ulcerated colorectal tumors may disrupt mucosal integrity and facilitate translocation of enteric organisms into the bloodstream. Several cohorts of patients with E. faecalis infective endocarditis have reported frequent colorectal lesions, including advanced adenomas and colorectal carcinoma [5-7]. However, a large nationwide study of first-time E. faecalis bloodstream infection found that, despite a higher relative risk of colorectal neoplasia than in matched controls, the absolute risk was insufficient to justify universal screening [8].

We report two cases of E. faecalis infective endocarditis in which gastrointestinal symptoms and cross-sectional imaging led to colonoscopy and the diagnosis of rectal adenocarcinoma. The cases illustrate the value of a targeted digestive evaluation and multidisciplinary collaboration between cardiology, infectious diseases, gastroenterology, radiology, pathology, and oncology.

Unlike population-level studies assessing the prevalence of colorectal abnormalities in patients with E. faecalis infection, these two cases provide direct multimodal documentation of the diagnostic pathway from infective endocarditis to colorectal imaging, colonoscopy, and histological confirmation of rectal adenocarcinoma. They illustrate how gastrointestinal symptoms and the absence of an alternative portal of entry may guide targeted colorectal investigation in routine clinical practice.

## Case presentation

Case 1

A 69-year-old man with a three-month history of unexplored chronic diarrhea was admitted with fever and an acute ischemic stroke. His temperature was 39 °C, and he was hemodynamically stable. Neurologic examination showed left-sided hemiplegia. Cardiovascular and peripheral examination identified an aortic systolic murmur, splenomegaly, Osler nodes, and subungual hemorrhages. Laboratory testing showed leukocytosis, anemia, and a markedly elevated C-reactive protein concentration of 359 mg/L.

**Figure 1:**
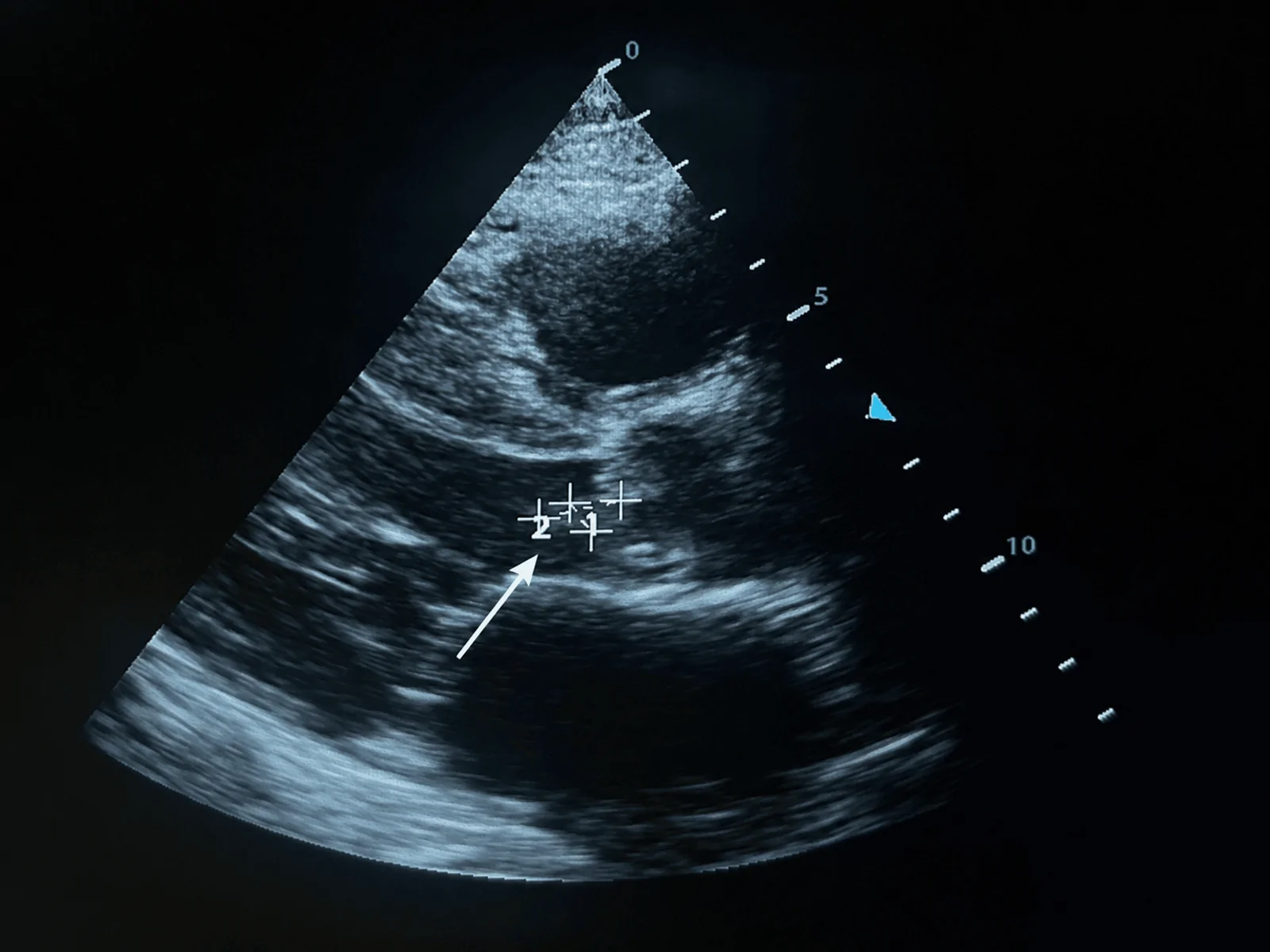
Transthoracic echocardiography showing mitral-valve vegetation in Case 1. A mobile mitral-valve vegetation measuring 12.5 × 5.6 mm is shown. The white arrow indicates the vegetation.

Brain computed tomography demonstrated an ischemic infarction in the posterior junctional territory. Transthoracic echocardiography revealed a mobile mitral-valve vegetation measuring 12.5 × 5.6 mm, with moderate mitral and aortic regurgitation and preserved left ventricular systolic function (Figure 1). Transesophageal echocardiography was not performed because of technical feasibility limitations and local unavailability. No valvular perforation, chordal rupture, or other destructive local complication was identified on transthoracic echocardiography; however, the absence of transesophageal imaging limited definitive assessment of additional local complications. Multiple blood cultures grew Enterococcus faecalis, establishing definite infective endocarditis. No urinary, biliary, catheter-related, or recent procedural source was identified.

The E. faecalis isolate was susceptible to vancomycin and gentamicin, and intravenous vancomycin plus gentamicin was administered for six weeks. Follow-up blood cultures became negative during antimicrobial therapy, although the exact date of clearance was not available in the medical record. During hospitalization, the patient developed findings compatible with immune-complex acute glomerulonephritis, associated with mild transient renal dysfunction. Renal function subsequently normalized. C-reactive protein decreased from 359 mg/L at admission to 60 mg/L during follow-up. Follow-up transthoracic echocardiography showed marked improvement after antimicrobial therapy, although quantitative post-treatment vegetation measurements were not available. Neurological recovery after the ischemic stroke was partial, and no infectious recurrence was documented during the available follow-up.

**Figure 2:**
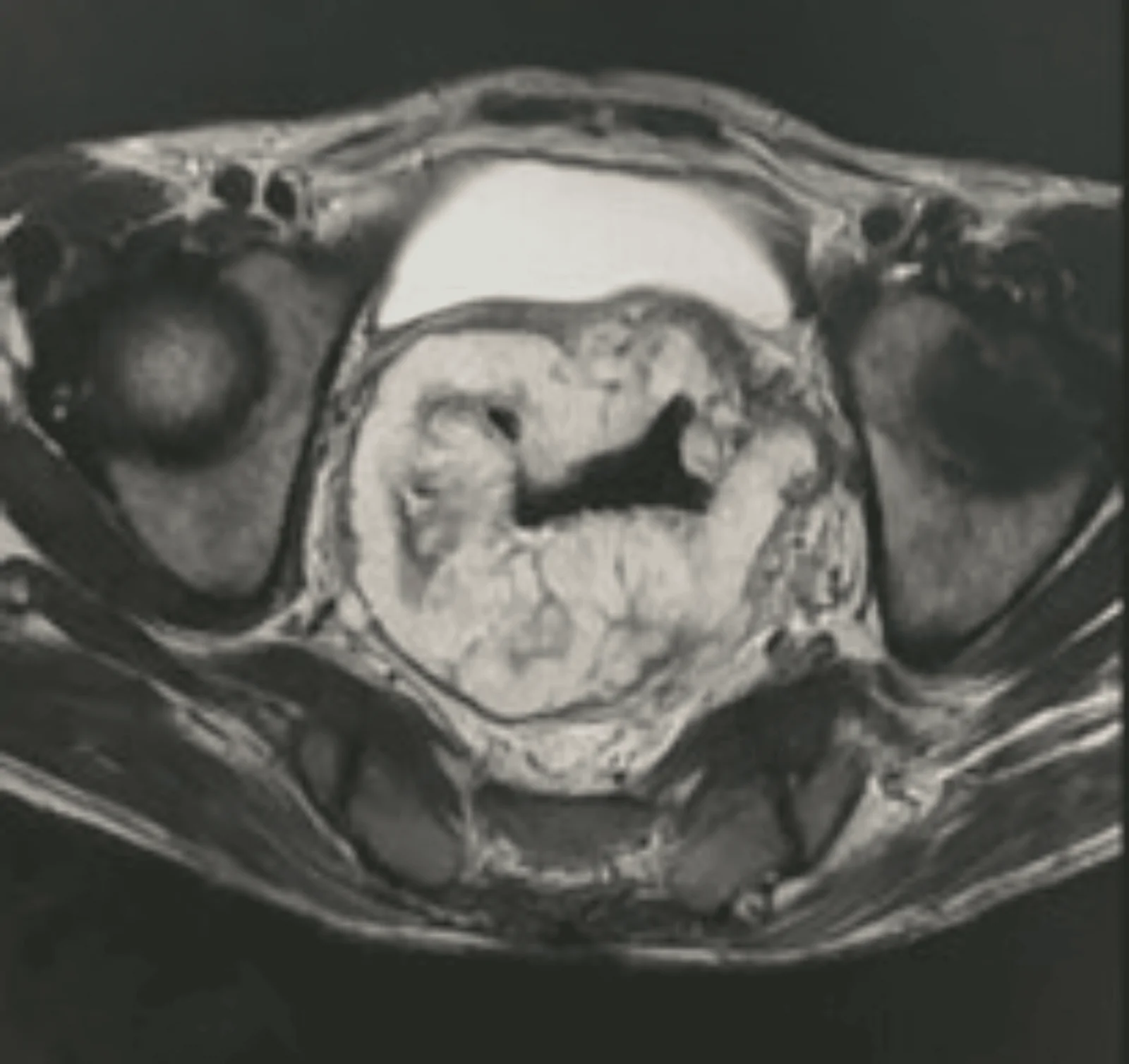
Pelvic magnetic resonance imaging showing a mid-rectal tumor in Case 1. Axial pelvic magnetic resonance image showing circumferential mid-rectal wall thickening with marked luminal narrowing, consistent with the subsequently histologically confirmed rectal adenocarcinoma.

Because of the chronic diarrhea, anemia, and absence of another portal of entry, a digestive evaluation was pursued. Pelvic magnetic resonance imaging demonstrated circumferential thickening of the mid-rectum with marked luminal narrowing (Figure 2).

**Figure 3:**
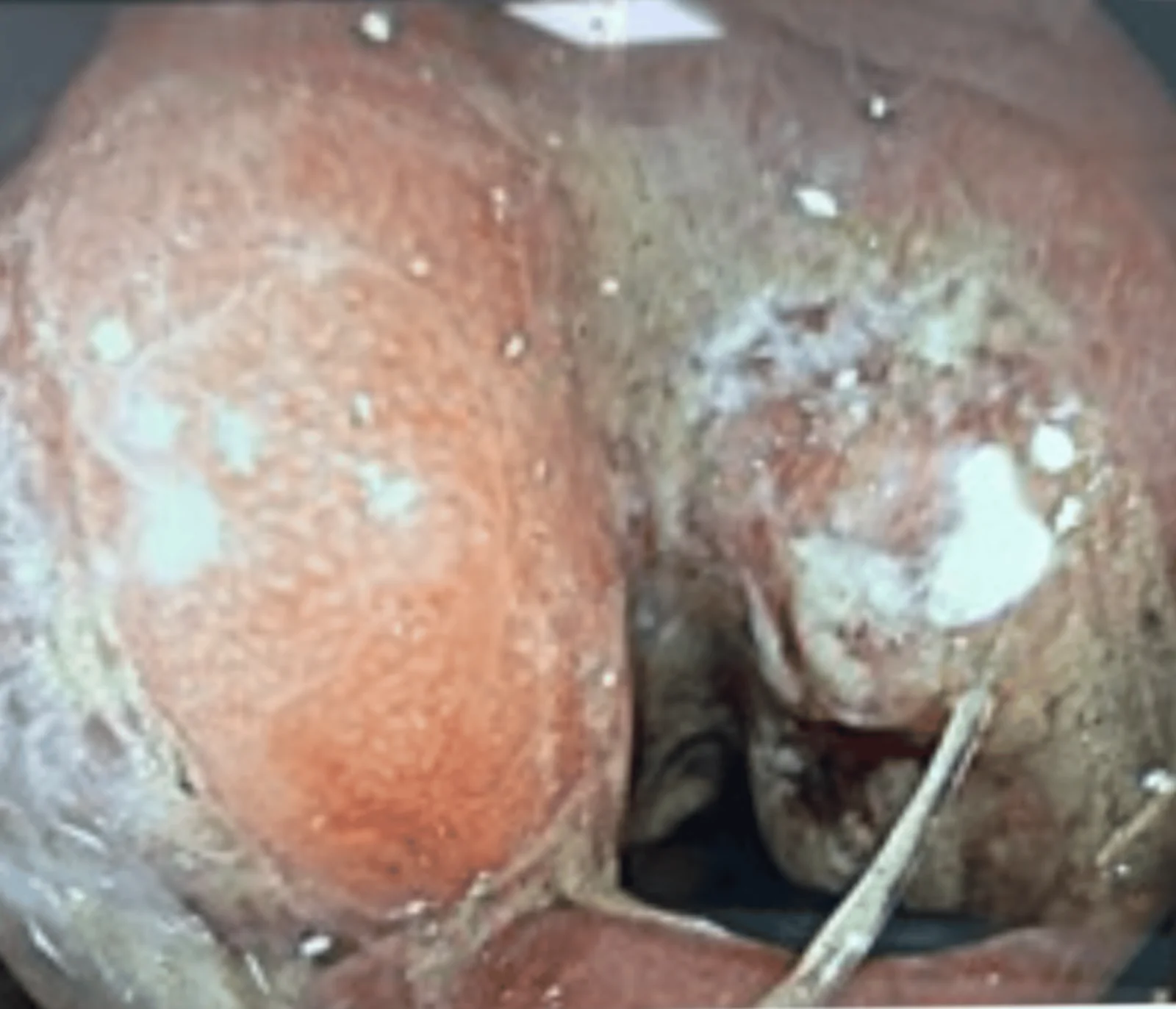
Colonoscopic appearance of the rectal tumor in Case 1. Colonoscopic image showing an ulcerated, exophytic rectal tumor occupying a substantial portion of the lumen and causing marked luminal narrowing.

**Figure 4:**
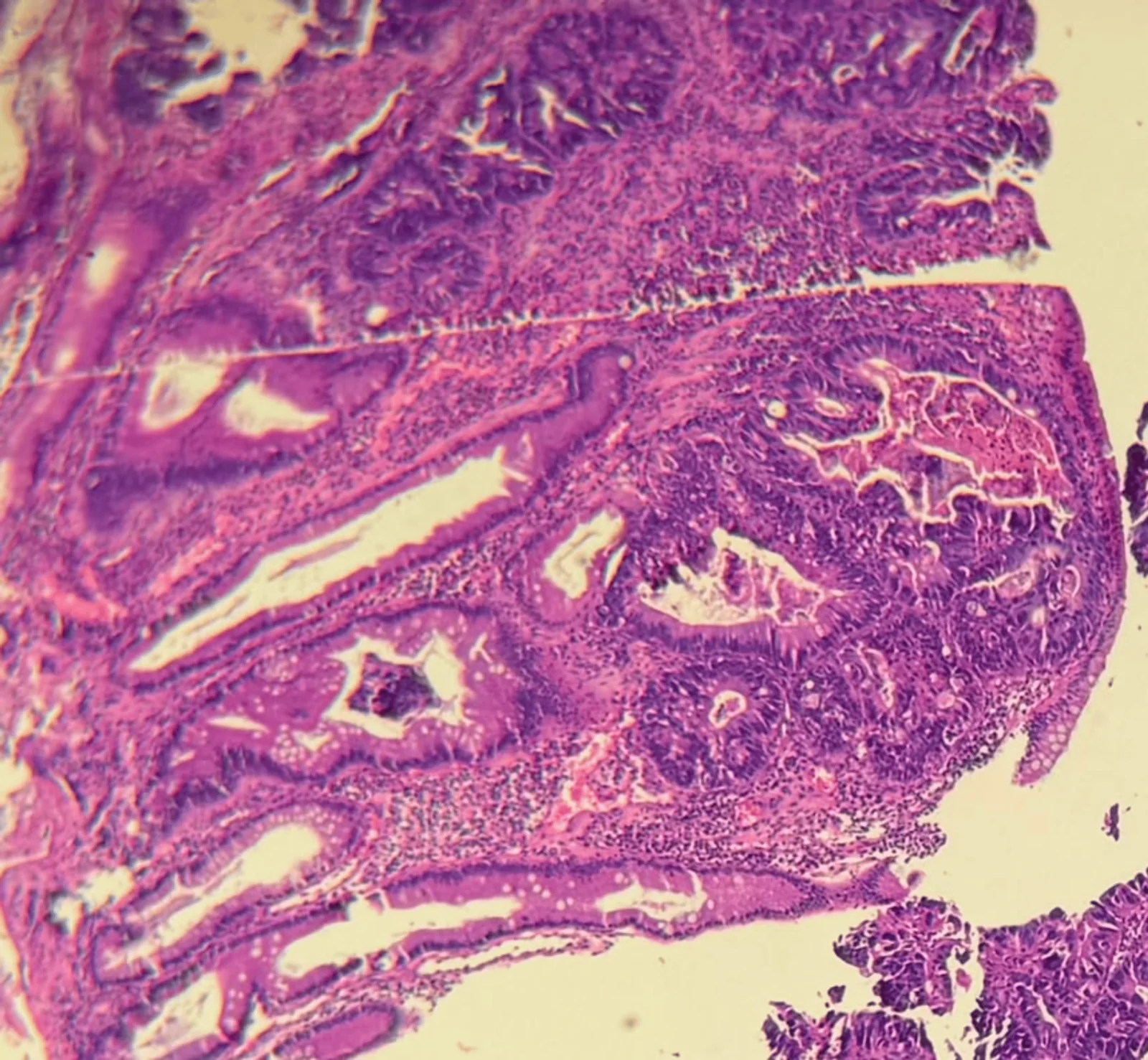
Histopathological findings of rectal adenocarcinoma in Case 1. Histopathological section showing infiltrative, irregular malignant glandular structures consistent with rectal adenocarcinoma.

Colonoscopy showed an ulcerated, exophytic rectal tumor causing marked luminal narrowing (Figure 3). Biopsy confirmed invasive rectal adenocarcinoma (Figure 4), and staging was consistent with cT4aN2b disease.

A protective colostomy was subsequently performed. The patient was discussed in a multidisciplinary setting involving cardiology, cardiovascular surgery, gastroenterology, and oncology. Cardiac surgical management was considered after stabilization of the infective endocarditis and was coordinated with the oncologic treatment strategy.

Case 2

A 40-year-old previously healthy man was admitted with an eight-day history of bloody diarrhea, fever, and polyarthritis. His temperature was 39 °C, and he was hemodynamically stable. Examination disclosed systolic murmurs over the mitral and aortic areas, splenomegaly, and arthritis of the small joints. Laboratory evaluation showed leukocytosis and a C-reactive protein concentration of 60 mg/L.

**Figure 5:**
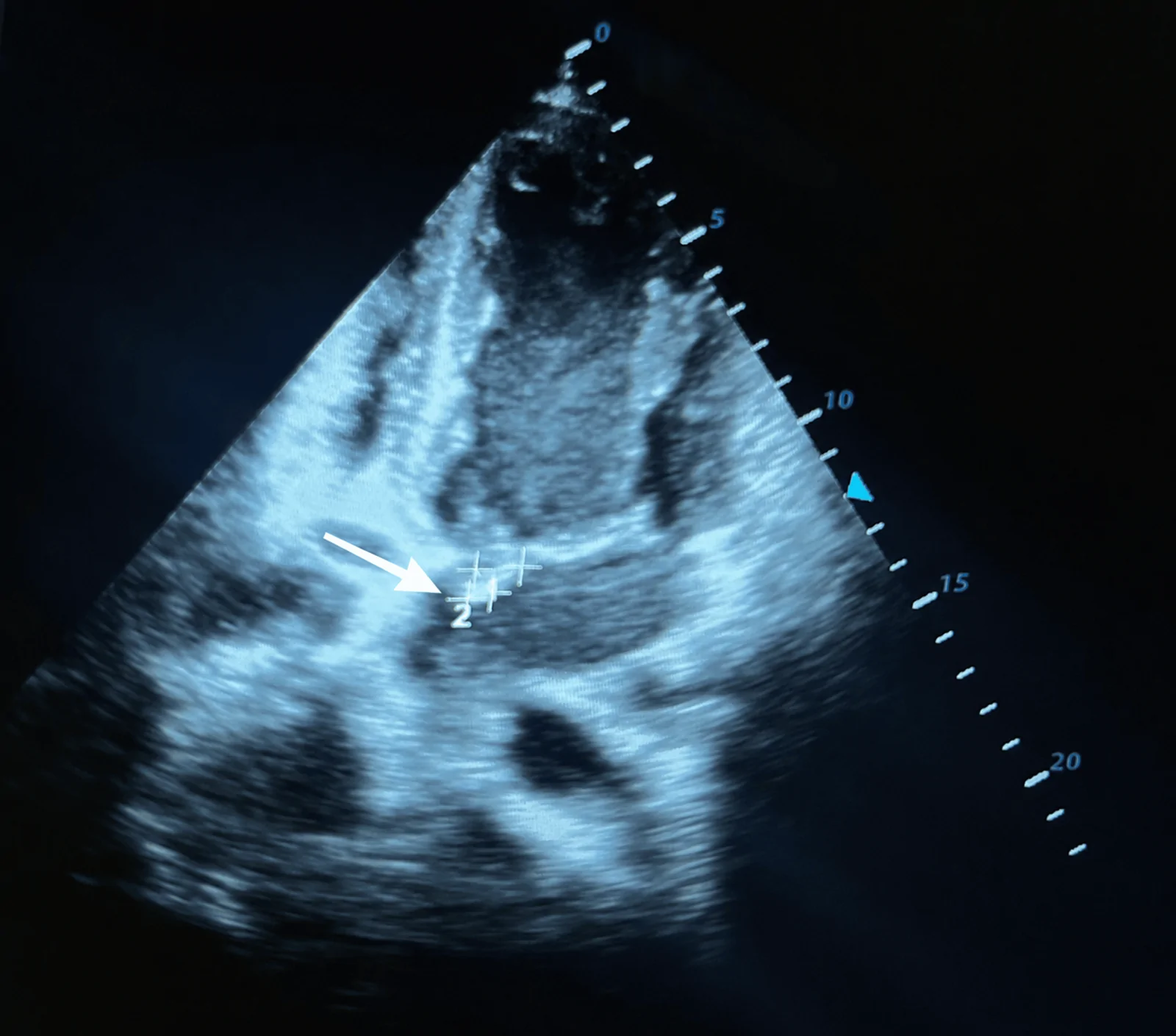
Transthoracic echocardiography showing aortic-valve vegetation in Case 2. A mobile aortic-valve vegetation measuring 13.2 × 6.5 mm is shown. The white arrow indicates the vegetation.

Transthoracic echocardiography identified a mobile aortic-valve vegetation measuring 13.2 × 6.5 mm, associated with severe aortic and mitral regurgitation and preserved left ventricular systolic function (Figure 5). The severe mitral regurgitation was considered pre-existing and functional rather than secondary to direct destructive mitral-valve involvement. No mitral vegetation, chordal rupture, or valvular perforation was identified on transthoracic echocardiography. Transesophageal echocardiography was not performed because of technical feasibility limitations and local unavailability; therefore, additional mitral involvement could not be definitively excluded. Blood cultures repeatedly yielded E. faecalis. No urinary, biliary, catheter-related, or recent procedural source was found.

**Figure 6:**
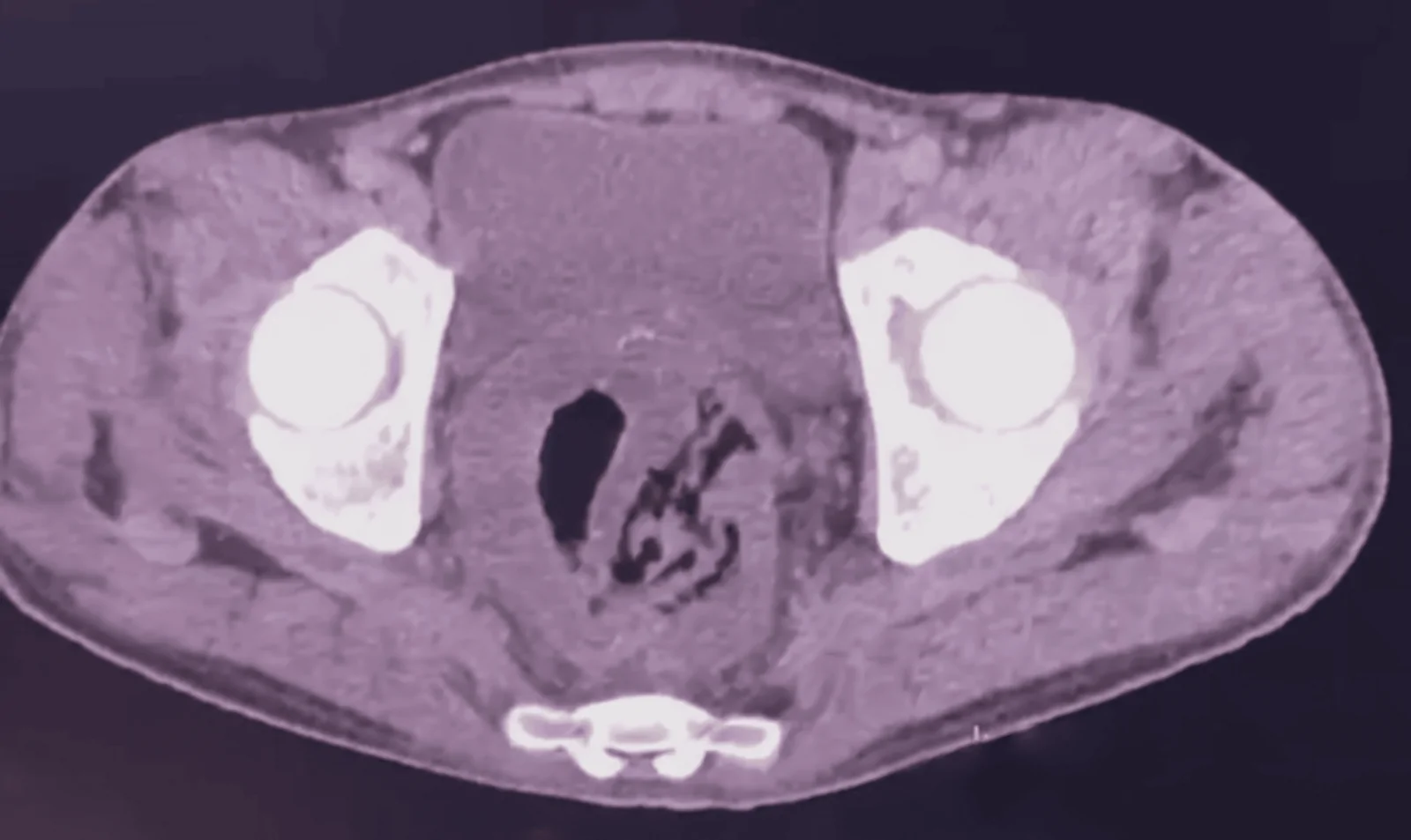
Axial abdominopelvic computed tomography image in Case 2. Axial abdominopelvic computed tomography image showing irregular circumferential thickening of the upper rectum and rectosigmoid junction with luminal narrowing.

The bloody diarrhea prompted early colorectal investigation. Abdominopelvic computed tomography showed irregular circumferential thickening of the upper rectum and rectosigmoid junction with luminal narrowing (Figure 6).

**Figure 7:**
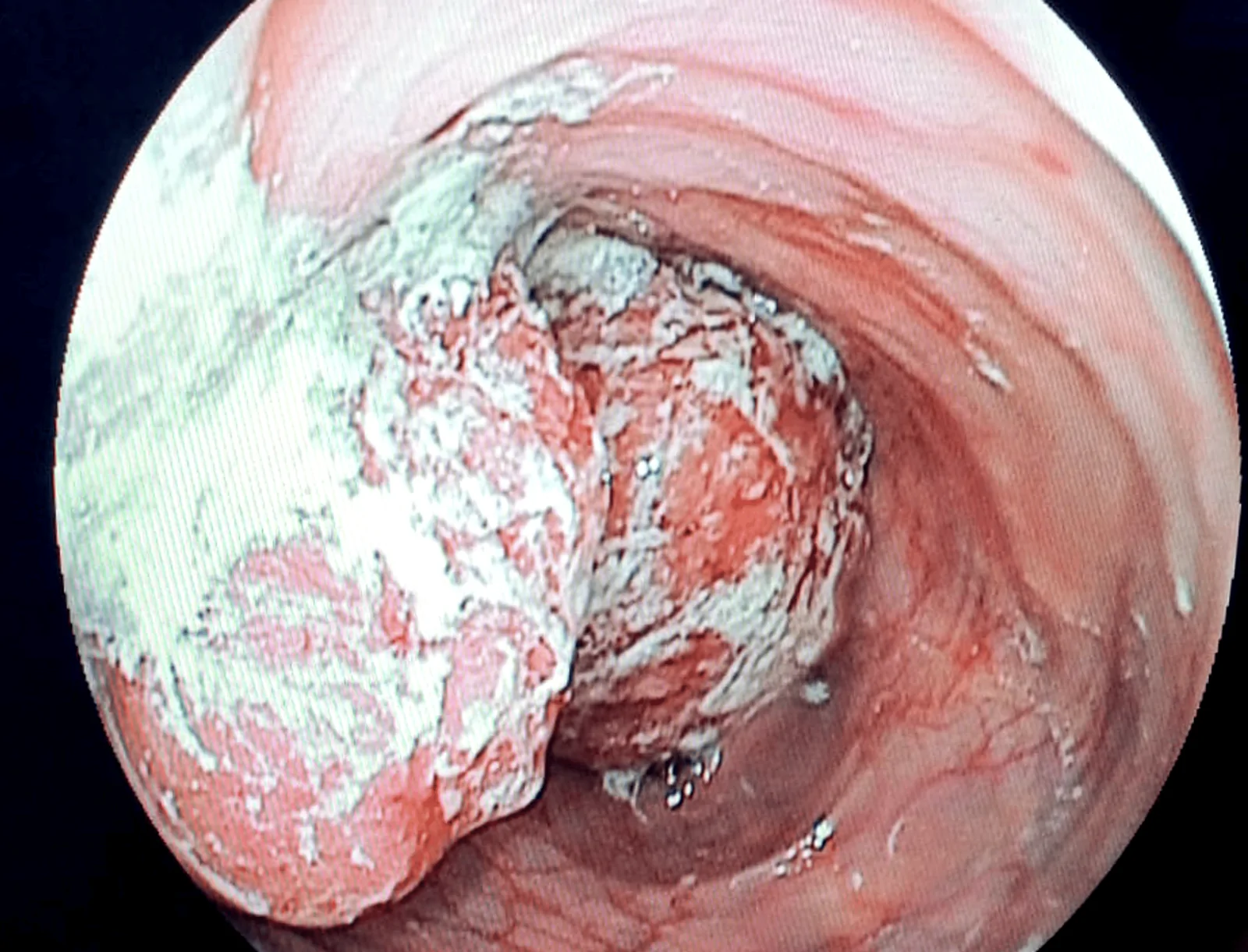
Colonoscopic appearance of an ulcerated fungating upper rectal tumor in Case 2. Colonoscopic image showing a large ulcerated, friable, exophytic rectal mass occupying much of the lumen and causing marked narrowing.

**Figure 8:**
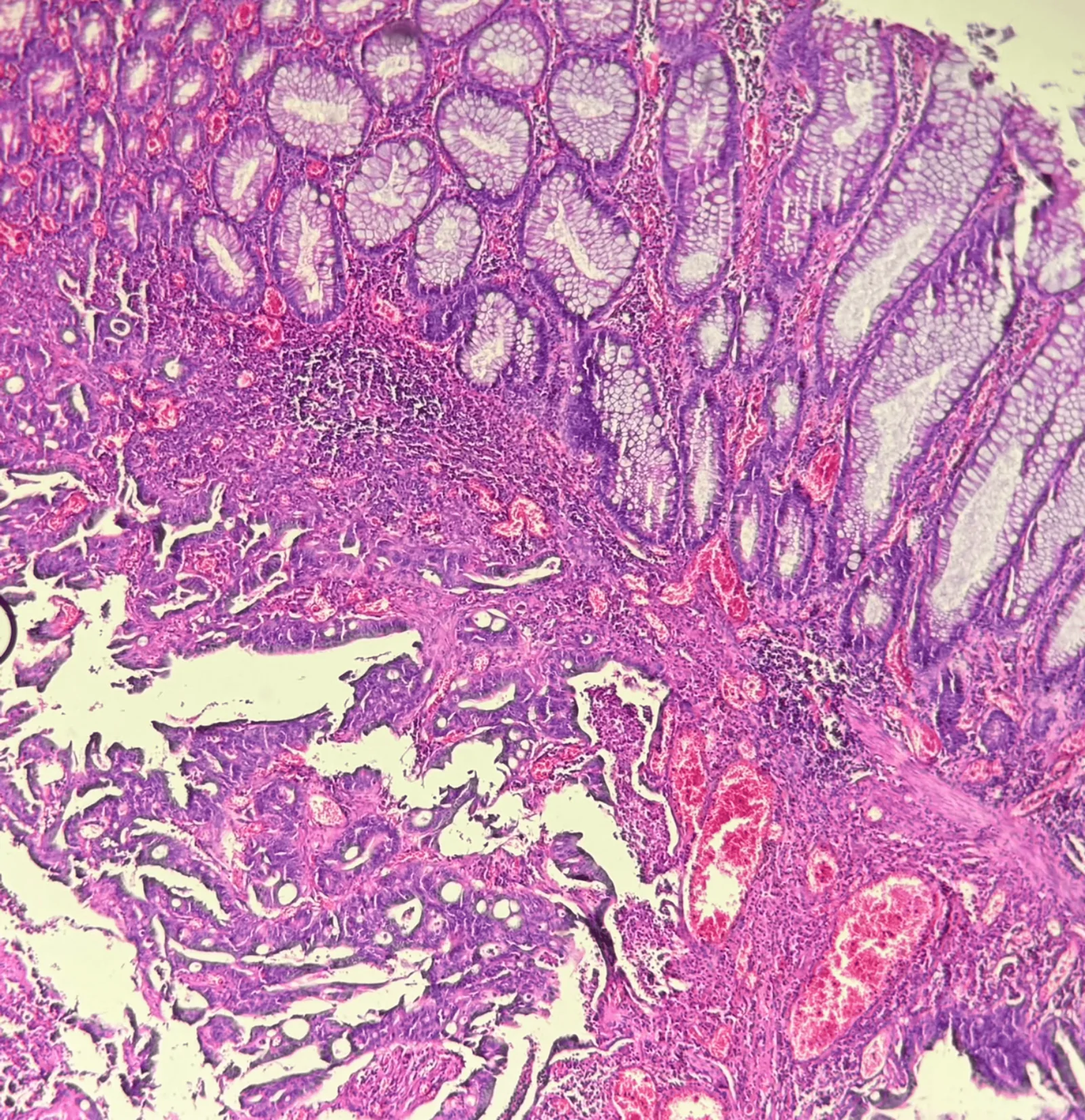
Histopathological findings in Case 2. Histopathological section showing rectal mucosa with infiltrative malignant glandular structures, consistent with adenocarcinoma. The complete pathological assessment established mucinous (colloid) differentiation.

Colonoscopy demonstrated a large ulcerated, friable, exophytic upper rectal mass occupying much of the lumen (Figure 7). Histopathological examination confirmed invasive mucinous (colloid) adenocarcinoma; the selected section shows infiltrative malignant glandular structures beneath the rectal mucosa (Figure 8).

The E. faecalis isolate was susceptible to vancomycin and gentamicin, and intravenous vancomycin plus gentamicin was administered for six weeks. Follow-up blood cultures became negative during antimicrobial therapy, although the exact date of clearance was not available in the medical record. C-reactive protein normalized during treatment. Follow-up transthoracic echocardiography showed marked improvement after antimicrobial therapy; quantitative post-treatment vegetation measurements were not available. The patient was discussed in a multidisciplinary setting involving cardiology, cardiovascular surgery, gastroenterology, and oncology. Cardiac surgical management was considered after stabilization of the infective endocarditis and was coordinated with management of the colorectal malignancy. The patient remained under regular multidisciplinary follow-up, initially every three months and subsequently every six months.

The principal clinical, echocardiographic, gastrointestinal, histopathological, and outcome findings of both cases are summarized in Table 1.

**Table 1:** Summary of the two cases. CRP, C-reactive protein; CT, computed tomography; MRI, magnetic resonance imaging; TEE, transesophageal echocardiography; TTE, transthoracic echocardiography; TNM, tumor-node-metastasis

Variable	Case 1	Case 2
Age/sex	69-year-old man	40-year-old man
Presentation	Fever, three-month history of chronic diarrhea, acute ischemic stroke	Fever, bloody diarrhea, polyarthritis
Initial CRP	359 mg/L	60 mg/L
Blood cultures	Enterococcus faecalis	Enterococcus faecalis
Antimicrobial susceptibility	Susceptible to vancomycin and gentamicin	Susceptible to vancomycin and gentamicin
Initial echocardiography	Mobile mitral-valve vegetation, 12.5 × 5.6 mm; moderate mitral and aortic regurgitation; preserved left ventricular systolic function	Mobile aortic-valve vegetation, 13.2 × 6.5 mm; severe aortic and mitral regurgitation; preserved left ventricular systolic function
TEE	Not performed because of technical feasibility limitations and local unavailability	Not performed because of technical feasibility limitations and local unavailability
Local valvular complications	No perforation, chordal rupture, or other destructive complication identified on TTE	No perforation or chordal rupture identified on TTE; severe mitral regurgitation considered pre-existing and functional
Antimicrobial treatment	Intravenous vancomycin plus gentamicin for six weeks	Intravenous vancomycin plus gentamicin for six weeks
Follow-up blood cultures	Became negative during treatment; exact date of clearance unavailable	Became negative during treatment; exact date of clearance unavailable
CRP evolution	Decreased from 359 to 60 mg/L	Normalized during treatment
Follow-up echocardiography	Marked improvement after antimicrobial therapy; quantitative post-treatment measurements unavailable	Marked improvement after antimicrobial therapy; quantitative post-treatment measurements unavailable
Other complications/outcomes	Mild transient renal dysfunction associated with immune-complex glomerulonephritis, with subsequent normalization of renal function; partial neurological recovery; no documented infectious recurrence	No documented infectious recurrence during the available follow-up
Digestive imaging	Pelvic MRI: circumferential mid-rectal wall thickening with marked luminal narrowing	Abdominopelvic CT: irregular circumferential thickening of the upper rectum and rectosigmoid junction with luminal narrowing
Colonoscopy	Ulcerated, exophytic rectal tumor causing marked luminal narrowing	Large ulcerated, friable, exophytic upper rectal mass occupying much of the lumen
Histopathology	Invasive rectal adenocarcinoma	Invasive mucinous (colloid) rectal adenocarcinoma
Tumor staging	cT4aN2b	Complete TNM staging not available
Oncologic management	Protective colostomy performed; subsequent oncologic management planned in a multidisciplinary setting.	Referred for oncologic management in a multidisciplinary setting
Cardiac surgical assessment	Discussed with cardiology and cardiovascular surgery; valvular intervention considered after stabilization of infective endocarditis.	Discussed with cardiology and cardiovascular surgery; valvular intervention considered after stabilization of infective endocarditis.
Follow-up	Multidisciplinary follow-up, initially every three months and subsequently every six months	Multidisciplinary follow-up, initially every three months and subsequently every six months

## Discussion

This two-case series illustrates the clinical relevance of considering E. faecalis infective endocarditis as a potential clue to underlying colorectal disease in appropriately selected patients. Both patients had gastrointestinal manifestations at presentation, together with no identified urinary, biliary, catheter-related, or recent procedural source of bacteremia. In this setting, cross-sectional abdominal imaging followed by colonoscopy was decisive in identifying rectal malignancy and obtaining histological confirmation. Importantly, these cases do not suggest that infective endocarditis was the first or sole manifestation of colorectal cancer, as both patients already had gastrointestinal symptoms. Rather, they illustrate how E. faecalis endocarditis can prompt a targeted search for a gastrointestinal portal of entry when clinical features support such an evaluation.

The cases also emphasize the potentially severe course of enterococcal endocarditis. Case 1 presented with an embolic ischemic stroke in the setting of a sizeable mitral-valve vegetation, whereas Case 2 had an aortic-valve vegetation associated with severe aortic and mitral regurgitation. Both isolates were susceptible to vancomycin and gentamicin, and both patients received six weeks of intravenous combination therapy. Follow-up blood cultures became negative during treatment, although the exact timing of clearance was not available from the medical records. In Case 1, C-reactive protein decreased from 359 to 60 mg/L, the mild renal dysfunction associated with immune-complex glomerulonephritis subsequently resolved, and neurological recovery was partial. In Case 2, C-reactive protein normalized. Follow-up transthoracic echocardiography demonstrated marked improvement in both patients, although quantitative post-treatment vegetation measurements were unavailable.

The cardiac severity of these presentations required multidisciplinary management. Both cases were discussed with cardiology and cardiovascular surgery, in coordination with gastroenterology and oncology. Given the embolic neurological complication and vegetation size in Case 1 and the significant valvular regurgitation in Case 2, valvular surgery was considered. The initial strategy was to complete antimicrobial therapy and achieve stabilization of the infective process before reassessing the timing of cardiac intervention and coordinating it with treatment of the newly diagnosed rectal malignancies. In Case 2, the severe mitral regurgitation was considered pre-existing and functional rather than the consequence of direct destructive mitral-valve involvement. No mitral vegetation, perforation, or chordal rupture was identified on transthoracic echocardiography. However, transesophageal echocardiography was not performed in either patient because of technical feasibility limitations and local unavailability; therefore, additional local complications or multivalvular involvement could not be definitively excluded.

The demographic characteristics of our patients partly reflect those reported in the literature. Both patients were men, consistent with the marked male predominance observed in large enterococcal endocarditis cohorts [3,4]. The 69-year-old patient closely matched the typical profile of an older male with enterococcal endocarditis, whereas the 40-year-old patient illustrates that clinically significant E. faecalis endocarditis associated with colorectal disease may also occur in younger adults. Enterococcal endocarditis remains a serious infection; large contemporary cohorts have reported substantial mortality and frequent cardiac and embolic complications [3,4].

Several observational studies support an association between E. faecalis infective endocarditis and colorectal disease. In a cohort of 154 patients, colorectal neoplasia was detected in 31 of 61 patients (50.8%) who underwent colonoscopy for E. faecalis infective endocarditis of unclear origin, including colorectal carcinoma in four patients (6.6%) [5]. In a multicenter observational study, 47 of 78 colonoscopies (60%) identified a colorectal lesion considered a potential source of bacteremia [6]. Similarly, in the GAMES cohort, colorectal disease was identified in 70.4% of the 142 patients who underwent colonoscopy, while advanced adenoma or colorectal carcinoma was found in 14.8% [7]. Individual case reports have also described E. faecalis endocarditis associated with previously undiagnosed colorectal adenocarcinoma [9,10].

These findings should nevertheless be interpreted cautiously. Colonoscopy was not systematically performed in all patients included in the observational cohorts, which introduces potential selection bias. Furthermore, many of these studies involved predominantly older patients with definite infective endocarditis and may not be generalizable to all patients with E. faecalis bacteremia. A 2025 nationwide study including 4,664 patients with first-time E. faecalis bloodstream infection reported six-month incidences of 0.45% for colorectal cancer and 2.34% for colorectal neoplasia. Although the relative risks were approximately three to four times higher than those in matched controls, the absolute risks remained low, leading the authors to conclude that these findings did not justify systematic colorectal screening of every patient with E. faecalis bloodstream infection [8].

A targeted rather than universal colonoscopic strategy therefore appears more appropriate. Colorectal evaluation should be strongly considered when E. faecalis bacteremia or endocarditis has no identifiable alternative source, is persistent or recurrent, or is accompanied by anemia, overt or occult gastrointestinal bleeding, altered bowel habits, unexplained weight loss, abdominal symptoms, or colorectal abnormalities on imaging. Age-appropriate colorectal cancer screening status should also be considered. In patients with severe infective endocarditis, the timing of bowel preparation and colonoscopy must be individualized according to hemodynamic stability, neurological status, and overall procedural risk.

The pathophysiological relationship between E. faecalis infection and colorectal neoplasia is biologically plausible but remains incompletely established. Ulcerated, friable, or stenosing colorectal tumors can disrupt epithelial barrier integrity and facilitate bacterial translocation into the bloodstream. Once bacteremia occurs, E. faecalis may adhere to damaged valvular tissue and persist through biofilm formation and other virulence mechanisms [1]. Experimental studies have also demonstrated that E. faecalis-derived reactive oxygen species can induce DNA damage and chromosomal instability in colonic epithelial cells [11,12]. These findings raise the possibility of a bidirectional interaction between E. faecalis and colorectal carcinogenesis; however, they do not establish a causal role for the organism in human colorectal cancer.

The comparison with Streptococcus gallolyticus is clinically relevant because the association between S. gallolyticus infection and colorectal neoplasia is well established [13]. The evidence supporting an analogous relationship for E. faecalis is less definitive. Accordingly, current evidence supports heightened clinical vigilance and selective colorectal investigation rather than considering the two microorganisms equivalent or recommending automatic colonoscopy after every episode of E. faecalis bacteremia.

The principal strength of this report is the concordant microbiological, echocardiographic, radiological, endoscopic, and histopathological documentation of two patients with E. faecalis infective endocarditis and rectal adenocarcinoma. The cases also illustrate the importance of multidisciplinary coordination when severe cardiac infection and newly diagnosed colorectal malignancy coexist. Several limitations should be acknowledged. This is a two-case series and therefore cannot estimate prevalence or establish causality. Molecular analysis linking bloodstream isolates directly to the colorectal tumors was not performed. Transesophageal echocardiography was unavailable, limiting detailed characterization of the affected valve leaflets and definitive exclusion of additional local or multivalvular complications. Exact dates of blood-culture clearance and quantitative measurements from follow-up echocardiography were also unavailable. Nevertheless, the concordant clinical findings support a pragmatic message: in patients with E. faecalis infective endocarditis and gastrointestinal symptoms or no alternative source of infection, targeted colorectal evaluation should be actively considered.

## Conclusions

E. faecalis infective endocarditis is a potentially serious infection that may provide an important diagnostic clue to underlying colorectal disease. In patients with gastrointestinal symptoms, anemia, bleeding, abnormal colorectal imaging, or no identifiable alternative portal of entry, targeted colorectal evaluation should be considered. In our two patients, colonoscopy was decisive in identifying rectal malignancy and obtaining histological confirmation.

These cases do not establish that infective endocarditis was the first or sole manifestation of colorectal cancer, as both patients already had gastrointestinal symptoms. They instead support a targeted, multidisciplinary approach integrating infectious disease, cardiology, cardiovascular surgery, gastroenterology, and oncology rather than universal colonoscopic screening after every E. faecalis bloodstream infection.

## References

[1] Madsen KT, Skov MN, Gill S, Kemp M (2017). Virulence factors associated with Enterococcus faecalis infective endocarditis: a mini review. Open Microbiol J.

[2] Delgado V, Ajmone Marsan N, de Waha S (2023). 2023 ESC Guidelines for the management of endocarditis. Eur Heart J.

[3] Chirouze C, Athan E, Alla F (2013). Enterococcal endocarditis in the beginning of the 21st century: analysis from the International Collaboration on Endocarditis-Prospective Cohort Study. Clin Microbiol Infect.

[4] Pericàs JM, Llopis J, Muñoz P (2020). A contemporary picture of enterococcal endocarditis. J Am Coll Cardiol.

[5] Pericàs JM, Corredoira J, Moreno A (2017). Relationship between Enterococcus faecalis infective endocarditis and colorectal neoplasm: preliminary results from a cohort of 154 patients. Rev Esp Cardiol (Engl Ed).

[6] Escolà-Vergé L, Peghin M, Givone F (2020). Prevalence of colorectal disease in Enterococcus faecalis infective endocarditis: results of an observational multicenter study. Rev Esp Cardiol (Engl Ed).

[7] Pericàs JM, Ambrosioni J, Muñoz P (2021). Prevalence of colorectal neoplasms among patients with Enterococcus faecalis endocarditis in the GAMES cohort (2008-2017). Mayo Clin Proc.

[8] Østergaard L, Dahl A, Chamat-Hedemand S (2025). Prevalence of colorectal cancer in patients with Enterococcus faecalis blood stream infection: a nationwide study. J Infect.

[9] Alziyadi A, Al Adwani M, Abdulaziz H, Aloufi R, Althagafi A, Alsulaimani A, Alotaibi A (2024). Enterococcus faecalis infective endocarditis associated with colorectal cancer. Cureus.

[10] Khan Z, Siddiqui N, Saif MW (2018). Enterococcus faecalis infective endocarditis and colorectal carcinoma: case of new association gaining ground. Gastroenterology Res.

[11] Huycke MM, Abrams V, Moore DR (2002). Enterococcus faecalis produces extracellular superoxide and hydrogen peroxide that damages colonic epithelial cell DNA. Carcinogenesis.

[12] Wang X, Allen TD, May RJ, Lightfoot S, Houchen CW, Huycke MM (2008). Enterococcus faecalis induces aneuploidy and tetraploidy in colonic epithelial cells through a bystander effect. Cancer Res.

[13] Boleij A, van Gelder MM, Swinkels DW, Tjalsma H (2011). Clinical Importance of Streptococcus gallolyticus infection among colorectal cancer patients: systematic review and meta-analysis. Clin Infect Dis.

